# Novel Bimetallic and Trimetallic Layered Double Hydroxides with the Compositions [Ni_6‑x_Zn_*x*_Al_3_(OH)_18_][A(H_2_O)_6_(SO_4_)_2_]·6H_2_O (*x* from 0 to 6; A= Na^+^ o K^+^)

**DOI:** 10.1021/acsomega.5c11618

**Published:** 2026-03-20

**Authors:** Anne Raquel Sotiles, Marco Tadeu Grassi, Mayara Padovan dos Santos, Gabriel Kavilhuka Metzger, Fernando Wypych

**Affiliations:** † Department of Chemistry Centro Politécnico, 28122 Federal University of Paraná, CP 19032, Jardim das Américas -, 81531-980 Curitiba, Paraná, Brazil; ‡ Post-Graduation Program in Engineering and Materials Science (PIPE) Centro Politécnico, Federal University of Paraná, CP 19032, Jardim das Américas -, 81531-980 Curitiba, Paraná, Brazil; § Post-Graduation Program in Materials Science and Engineering (PPGCEM), Federal University of Technology, CEP 86036-370 Londrina, Paraná, Brazil

## Abstract

Bimetallic and trimetallic layered double hydroxides with the composition [Ni_6‑*x*_Zn_*x*_Al_3_(OH)_18_]­[A­(H_2_O)_6_(SO_4_)_2_]·6H_2_O (*x* from 0 to 6; A = Na^+^ or K^+^) were synthesized by coprecipitation with increased pH and characterized by several instrumental techniques. X-ray diffraction (XRD) indicated the prevalence of basal diffraction peaks with basal distances close to 11 Å, typical of LDHs intercalated with sulfate in double-layer arrangement in the presence of potassium cations, in both cases hydrated. Fourier-transform infrared spectroscopy (FTIR) revealed a typical series of bands, attributed to S–O (from sulfate), M–OH (M = metal of the layers), and O–H (from water molecules and hydroxide layers). Scanning electron microscopy (SEM) images indicated the typical platelet-like morphology of LDH, with micrometric particles having nanometric thicknesses, while energy-dispersive X-ray spectroscopy (EDS) indicated the expected distribution of metals in the series. Inductively coupled plasma optical emission spectroscopy (ICP-OES) confirmed the compositions of the materials, which were close to ideal according to the chemical used in the syntheses, suggesting the absence of simultaneous precipitation of other materials (especially hydroxides). Thermogravimetric analyses (TGA) and the respective derivative curves (DTG) also confirmed the decomposition in several steps, attributed to the removal of physiosorbed water molecules, dehydration of cations/anions, dehydroxylation of the layers, and decomposition of the metal sulfates to oxides/spinels.

## Graphical abstract

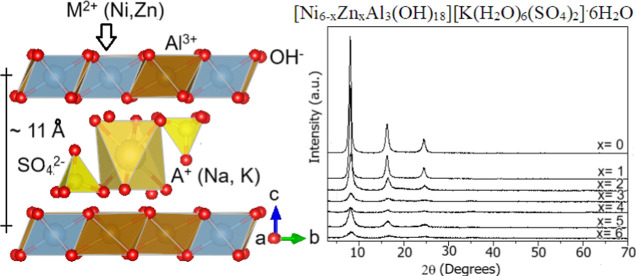

## Introduction

Materials chemistry is currently recognized as one of the most important branches of science due to the strong possibility of discovering new compounds and applications in the most diverse areas. Layered or two-dimensional materials (2D) are in the forefront of materials research due to the vast number of members of this family, which increase every day with attractive physical and chemical properties. Among the promising materials are layered double hydroxides (LDHs), which have been widely studied and present surprising results in fundamental science and technological applications across different fields. This is due to their compositional variability, controllable particle sizes, possibility of being delaminated/exfoliated, easy and cost-effective synthesis, and tailored properties.^1–7^

LDHs are compounds whose structures are derived from brucite (Mg­(OH)_2_), consisting of divalent cations that occupy octahedral centers coordinated with hydroxyl anions, which share edges to build two-dimensional trioctahedral layers. In LDHs, some of the divalent cations are replaced by trivalent cations, resulting in an excess of positive charges in the layers, which need to be balanced by the intercalation of anions (normally hydrated).

Traditional LDHs have the formula M^2+^_1–*x*_M^3+^_*x*_(OH)_2_(A^–*n*^)_*x*/*n*_·*y*H_2_O and have varied properties depending on the constituent metals in the layers and the proportion between them, in addition to the anions intercalated in the interlayer space (again normally hydrated).

In addition to traditional LDHs, there are also aluminum-rich LDHs, which can be considered derivatives of gibbsite (Al­(OH)_3_), where the Al^3+^ anions are octahedrally coordinated with six hydroxyls sharing edges, with only two-thirds of the Al­(OH)_6_ octahedra occupying the dioctahedral layers of the structure and one-third of the octahedral sites remaining vacant. When solid Al­(OH)_3_ reacts with lithium salts, Li^+^ cations occupy the vacant octahedral sites and anions are intercalated to compensate for the excess positive charges, obtaining LDHs with chemical compositions [Li­(Al­(OH)_3_)_2_]­(A^*n*–^)_1/*n*_·*y*H_2_O.^8^

In addition to the nontraditional LDHs, a series of compounds with the chemical composition [M^2+^_6_Al_3_(OH)_18_]­[Na­(H_2_O)_6_(SO_4_)_2_]·6H_2_O have already been described as occurring in the form of minerals. Shigaite is one of these substances, with the chemical composition [Mn_6_Al_3_(OH)_18_]­[Na­(H_2_O)_6_(SO_4_)_2_]·6H_2_O, having layers like the hydrotalcite (Mg_6_Al_2_(OH)_16_CO_3_·4H_2_O) but with unusual interlayer species, consisting of a cluster with the chemical composition ([Na­(H_2_O)_6_(SO_4_)_2_]·6H_2_O).^3–9^ Other members in the group are natroglaucocerinite ([Zn_6_Al_3_(OH)_18_]­[Na­(H_2_O)_6_(SO_4_)_2_]·6H_2_O), motukoreaite ([Mg_6_Al_3_(OH)_18_]­[Na­(H_2_O)_6_(SO_4_)_2_]·6H_2_O), nikischerite ([Fe^2+^_6_Fe^3+^_3_(OH)_18_]­[Na­(H_2_O)_6_ (SO_4_)_2_]·6H_2_O), and even the series of green rusts.^10–14^

This new layered double hydroxide falls outside the nomenclature of anionic clay since it can exchange cations, anions, and even both simultaneously. Most of these compounds were recently synthesized and new members were included in the list, like U-phase, [3Ca_2_Al­(OH)_6_]­[Na­(H_2_O)_6_(SO_4_)_2_·6H_2_O],^15^ and others obtained by exchanging sodium with other alkali metal cations like lithium and potassium,^16–18^ and sulfate with monohydrogen phosphate.^19^ The synthesis and characterization of trimetallic LDHs from this nontraditional family, involving several metals like zinc, cobalt, manganese, copper, and aluminum, were also recently described.^20,21^

In the present study, we report a new series of bimetallic and trimetallic LDHs with the composition [Ni_6–*x*_Zn_*x*_Al_3_(OH)_18_]­[A­(H_2_O)_6_(SO_4_)_2_]·6H_2_O (*x* from 0 to 6; A = Na^+^ or K^+^), which were synthesized by coprecipitation with increased pH and fully characterized by various instrumental techniques. Besides the pH, other factors need to be considered during the precipitation, which will influence the size of the particles, morphology, and crystallinity. The nucleation and crystal growth play a decisive factor in the crystal quality and in general very slow addition of the precipitating agent, which needs to be in low concentration (as well as the metal solution), the stirring that needs to be vigorous, the temperature of the precipitation that needs to be higher as possible, and also the time the mixture will stay for crystals ripening. All these factors are necessary for the crystals’ improved quality and stabilization of larger particles.

We would like to point out that although the so-called trimetallic shigaite-type compounds, as in the present case ([Ni_6–*x*_Zn_*x*_Al_3_(OH)_18_]­[A­(H_2_O)_6_(SO_4_)_2_]·6H_2_O), have four metals (three in the layers and one intercalated alkali metal), in order to avoid conflict with the trimetallic hydrotalcite-like compounds, only the metals present in the layers are considered.

## Materials and Methods

All chemicals were of analytical grade and used without further purification: Al_2_(SO_4_)_3_·16H_2_O—Reatec 98–102%; ZnSO_4_·7H_2_O—Reatec 99%; NiSO_4_·7H_2_O—Sigma Adrich 99%, KOH—Dinâmica 85%, K_2_SO_4_—Reatec 99%, NaOH—Êxodo 97%; and Na_2_SO_4_—Neon 99%. To prepare the solutions, ultrapure and decarbonated water from a Milli-Q system (18.2 MΩ cm at 25 °C, Millipore Simplicity UV, Bedford, USA) was used. To remove carbonate, water was boiled and cooled using a constant flow of commercial nitrogen. A total of 100 mL of the solutions containing metal salts and the source of potassium (K_2_SO_4_) or sodium (Na_2_SO_4_) were added to 1 L glass reactors. The temperature was then raised to 90 °C and the titration occurred by using KOH or NaOH, both 1 mol/L. The alkaline solution was dropwise added to the stirred solution inside the reactor using a peristaltic pump. Commercial nitrogen was continuously supplied to the reactor after the gas was filtered with a solution of NaOH to remove CO_2_. The content of the reactor was transferred to a closed glass container that was hydrothermally treated in an oven at 90 °C for 120 h. To separate the solids, the suspension was centrifuged at 4000 rpm for 5 min, redispersed and washed five times with decarbonated water and dried at 60 °C for 48 h.

The method of synthesis by coprecipitation followed by hydrothermal treatment adopted in the present work was the best method with reproducibility of results and materials of better crystallinity compared to other methods investigated in optimization experiments.

X-ray diffraction (XRD) patterns were obtained by placing the slurry after the last centrifugation into glass holders, which were dried at room temperature. The analyses were performed with a Shimadzu XRD-6000 diffractometer using Cu Kα = 1.5418 Å radiation, operating with a current of 30 mA, voltage of 40 kV, and dwell time of 2° min^–1^.

Fourier-transform infrared (FTIR) spectra were obtained using spectroscopic KBr pellets containing about 1% of the sample, which were gently crushed and pressed at 5 tons. The measurements were obtained in transmission mode using a Bruker Vertex70 spectrophotometer, and the spectra were collected from 400 to 4000 cm^–1^, with a resolution of 2 cm^–1^ and accumulation of 32 scans.

Scanning electron microscopy (SEM) and energy-dispersive spectroscopy (EDS) measurements were performed by using a Tescan Vega3LMU microscope with AZ Tech software. The samples were deposited on copper tapes and after the EDS analyses were sputtered with a thin gold layer to obtain the SEM images.

The chemical analyses were performed by inductively coupled plasma-optical emission spectrometry (ICP-OES) using a Thermo Fisher Scientific spectrometer (model iCAP 6500) with axial view and Thermo Fisher Scientific iTeVa software, version 1.2.0.30. The samples were dissolved in 1.0% v/v HNO_3_ using Milli-Q water solutions and the data were collected in duplicate and average values were used, with the standard deviation of around 2%.

Thermogravimetric analyses (TGA) were performed using a PerkinElmer TGA 4000 in the range of room temperature to 1000 °C, under a synthetic air atmosphere with a flow rate of 50 mL/min. The samples were placed in alumina crucibles, and the curves were obtained at a heating rate of 10 °C/min.

## Results and Discussion

Tables 1 and 2 show the amounts of the chemicals used during the synthesis (in millimoles), along with the initial and final pH value after the precipitation.

**Table 1:** Amounts of Chemicals Used (in mmol) and the Initial and Final pH in the Ni/Zn/Al–K LDH Syntheses

system	NiSO_4_	ZnSO_4_	Al_2_(SO_4_)_3_	K_2_SO_4_	initial pH	final pH
6Ni/3Al–K	25.285	-	6.323	2.587	3.41	7.39
5Ni/1Zn–3Al–K	20.958	4.190	6.288	2.568	3.82	7.52
4Ni/2Zn–3Al–K	17.055	8.333	6.254	2.544	3.80	7.54
3Ni/3Zn–3Al–K	12.433	12.435	6.216	2.546	3.93	7.49
2Ni/4Zn–3Al–K	8.242	16.487	6.184	2.531	3.78	7.58
1Ni/5Zn–3Al–K	4.100	20.496	6.150	2.516	3.85	7.49
6Zn/3Al–K	-	24.454	6.116	2.507	3.33	8.65

**Table 2:** Amounts of Chemicals Used (in mmol) and the Initial and Final pH in the Ni/Zn–Al–Na LDH Syntheses

system	NiSO_4_	ZnSO_4_	Al_2_(SO_4_)_3_	Na_2_SO_4_	initial pH	final pH
6Ni/3Al–Na	25.639	-	6.409	2.142	3.43	7.54
5Ni/1Zn–3Al–Na	19.880	4.249	6.327	2.120	3.97	7.56
4Ni/2Zn–3Al–Na	16.896	8.449	6.337	2.113	3.93	7.55
3Ni/3Zn–3Al–Na	12.601	12.602	6.301	2.100	3.91	7.53
2Ni/4Zn–3Al–Na	8.353	16.708	6.266	2.084	3.87	7.55
1Ni/5Zn–3Al–Na	4.143	20.770	6.231	2.077	3.82	7.52
6Zn/3Al–Na	-	24.799	6.209	2.070	3.84	9.49

These concentrations, if all the elements had been incorporated into the structure, would have had ideal stoichiometries of bimetallic phases of Ni_6_Al_3_(OH)_18_]­[A­(H_2_O)_6_(SO_4_)_2_]·6H_2_O and [Zn_6_Al_3_(OH)_18_]­[A­(H_2_O)_6_(SO_4_)_2_]·6H_2_O and trimetallic phases of [Ni_5_Zn_1_Al_3_(OH)_18_]­[A­(H_2_O)_6_(SO_4_)_2_]·6H_2_O, [Ni_4_Zn_2_Al_3_(OH)_18_]­[A­(H_2_O)_6_(SO_4_)_2_]·6H_2_O, [Ni_3_Zn_3_Al_3_(OH)_18_]­[A­(H_2_O)_6_(SO_4_)_2_]·6H_2_O, [Ni_2_Zn_4_Al_3_(OH)_18_]­[A­(H_2_O)_6_(SO_4_)_2_]·6H_2_O, and [NiZn_5_Al_3_(OH)_18_]­[A­(H_2_O)_6_(SO_4_)_2_]·6H_2_O, where A = Na^+^ or K^+^.

All the XRD patterns from both series of samples (Figures 1 and 2) indicated only basal peaks, due to the orientation of the layered particles on the sample holders, exposing the particles’ basal planes.

The basal distances were calculated using the higher order basal peaks since the error in this determination is higher when the first peak is used. No impurities were detected in any of the samples, suggesting that all elements used in the synthesis were incorporated into the samples.

The XRD patterns of all the samples from the Zn/Ni–Al–K series (Figure 1) presented a series of basal peaks with basal distance close to 11 Å, typical of samples intercalated with sulfate in a double-layer arrangement in the presence of potassium, in all cases hydrated.^9,21,22^

**Figure 1:**
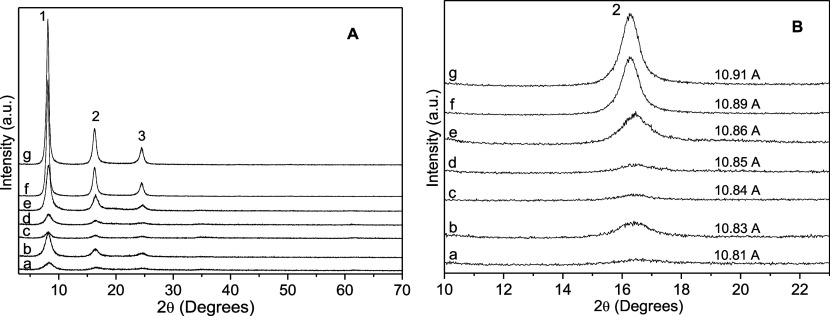
(A) X-ray diffraction patterns of the Zn/Ni–Al–K system. 6Ni/3Al–K (a), 5Ni/1Zn–3Al–K (b), 4Ni/2Zn–3Al–K (c), 3Ni/3Zn–3Al–K (d), 2Ni/4Zn–3Al–K (e), 1Ni/5Zn–3Al–K (f), and 6Zn/3Al–K (g). (B) XRD expansion in the region of the second basal peak.

The basal distance of 10.91 Å attributed to the synthetic Zn/Al–K phase varied slightly in different batches of synthesis, being previously reported as 11.18 or 11.40 Å.^17^ It is important to mention that sulfate intercalated in traditional LDHs can present different basal distances (from 7 Å in the dehydrated form to 11 Å in the fully hydrated form).

In traditional LDHs, this variation of the basal distances is highly dependent on the humidity, pressure, and temperature.^22,23^ Also, the basal distances are calculated from only one broad basal peak. This peak is strongly influenced by the quality of the film positioned in the sample holder, which can suffer small peak shifting due to the variable thickness of these films, directly impacting the determination of the basal distances.

In the Zn/Ni–Al–K series, the basal distance increased slightly when nickel was substituted by zinc in the system Zn/Al–Ni–K, which agrees with the increase of the ionic radii from Ni^2+^ (0.704 Å) in relation to Zn^2+^ (0.744 Å), all having the same coordination number,^24^ suggesting the system followed Vegard’s law.^25^ The presence of different cations in the layers did not interfere with the interaction with the intercalated [K­(H_2_O)_6_(SO_4_)_2_·6H_2_O]^3–^ cluster.

Due probably to the disorder in the interlayer region, the phase 6Ni/3Al–K (Figure 1A,B-a) had exceptionally low crystallinity, which increased in the series, culminating with the best crystallinity in the sample 6Zn/3Al–K (Figure 1A,B-g). Also, in the Ni/Zn–Al–K series, due to the broadening of the diffraction’s peaks, it can be inferred that the number of packed layers along the basal axis increased when Ni was replaced by Zn.

According to the Scherrer equation (*D*_p_ = (0.94 × λ)/(β × Cos θ), where *D*_p_ = average particle size, β = line broadening in radians, θ = Bragg angle, λ = X-ray wavelength), the sizes of the particles along the basal directions were estimated to be ca. 8 nm in the sample 6Ni/3Al–K (Figure 1A,B-a) and 13 nm in the sample 6Zn/3Al–K (Figure 1A,B-g). This gave estimates of the number of packed layers along this direction to be from 8 to 12.

In the Zn/Ni–Al–Na series (Figure 2), a similar behavior was observed, where the phase containing only nickel (Figure 2A,B-a) had the lowest crystallinity, which increased with the incorporation of zinc (Figure 2A,B-g). In sample 6Zn/3Al–Na (Figure 2A,B-g), the particles along the basal direction were estimated to be 35 nm, giving 32 packed layers.

**Figure 2:**
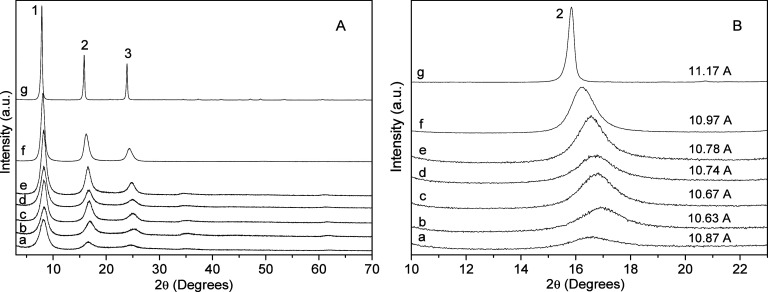
A) X-ray diffraction patterns of the Zn/Ni–Al–Na system. 6Ni/3Al–Na (a), 5Ni/1Zn–3Al–Na (b), 4Ni/2Zn–3Al–Na (c), 3Ni/3Zn–3Al–Na (d), 2Ni/4Zn–3Al–Na (e), 1Ni/5Zn–3Al–Na (f), and 6Zn/3Al–Na (g). (B) XRD expansion in the region of the second basal peak.

The basal distance of 11.17 Å attributed to the synthetic 6Zn/3Al–Na phase agrees well with the basal distance of the mineral natroglaucocerinite (11.18 Å)^10^ and to the synthetic one (11.14 Å).^17^ Except for 6Ni/3Al–Na (Figure 2A,B-a), a similar behavior was observed in the basal distance to that in the phase intercalated with sulfate and potassium, which grew from nickel to zinc in the series. However, here the effect of increasing the basal distance was more pronounced, indicating that the ionic radii of the metals in the layers are not the only effect to determine the basal distance but also the interaction of the layers with the intercalated cluster [Na­(H_2_O)_6_(SO_4_)_2_·6H_2_O]^3–^. This interaction was weaker in sample 6Zn/3Al–Na (Figure 2A,B-g) than in sample 6Ni/3Al–Na (Figure 2A,B-a). Since the ionic radii of Zn and Ni are almost the same and the basal distance is the summation of the layers’ thickness and the size of the intercalated clusters, which are composed of hydrated sulfate in double layer configurations and the ionic radii of the hydrated alkali metal cations, using the geometric consideration in the structure of shigaite (Na–O distance of 2.471 Å and Mn in the layers to Na cations = 5.449 Å) plus the layers’ thickness close to 4.72 Å, the value obtained is 10.89 Å (shigaite basal distance of 11.02 Å).^9^ The basal distance can basically be attributed to the space occupied by the water hexacoordinated alkali metal cations and the interaction of the triple-charged cluster with the positively charged layers.^26^

The FTIR spectra of all samples from the Zn/Ni–Al–K series (Figure 3A) are characterized by the presence of typical bands of S–O ν_3_ asymmetrical bending vibrations of sulfate in the region 1100 cm^–1^. The broad ν_3_ band and the presence of a ν_1_ band (close to 960 cm^–1^) and ν_4_ bands (close to 600 cm^–1^) suggest that sulfate is allocated in a distorted tetrahedral environment, interacting with the layers, water molecules, and potassium cations.^18,27–29^

**Figure 3:**
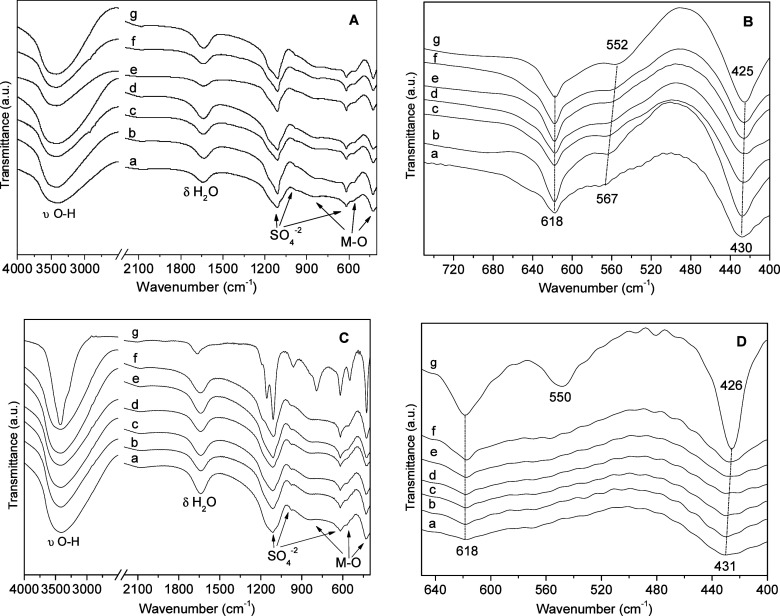
FTIR spectra of the Zn/Ni–Al–K (A,B) and Zn/Ni–Al–Na (C,D) systems. 6Ni/3Al (a), 5Ni/1Zn–3Al (b), 4Ni/2Zn–3Al (c), 3Ni/3Zn–3Al (d), 2Ni/4Zn–3Al (e), 1Ni/5Zn–3Al (f), and 6Zn/3Al (g).

The broad band in the region of 3470 cm^–1^ is attributed to stretching vibrations of structural O–H and intercalated/physiosorbed water molecules, while the band close to 1600 cm^–1^ is attributed to water molecule bending. The only clear distinction in this region is attributed to the narrowing of the band in the most crystalline sample of 6Zn/3Al–Na (Figure 3C-g), which can be attributed to the restricted vibration of the O–H groups, related to improved crystallinity and interaction between the cationic layers and the anionic clusters. As reported previously, the narrow band in the region of 3600 cm^–1^ can be attributed to Zn–OH and Ni–OH bonds in the octahedral layers.^30^

Due to the very similar ionic radii of nickel and zinc, the M–OH and M–O–M (M = Al, Zi, Ni) bands occur very close, but some displacements can be noticed (Figure 3 C,D), in agreement with the M–O bond distances (Al–O = 1.90; Ni–O = 2.07; and Zn–O = 2.11).^24^ Based on the same logic, the bigger displacement is observed for the band in the region of 550–570 cm^–1^, which occurs at 552 cm^–1^ for 6Zn/3Al (Figure 3B-g) and 567 cm^–1^ for 6Ni/3Al (Figure 3B-a).

Smaller M–O distances present higher energies in the vibrations, and consequently, the bands occur at higher wavenumbers. These bands are broad due to the coalescence of various M–OH and M–O–M bonds present in the layers’ structures.

The series Zn/Ni–Al–Na presents very similar behavior except for the sample 6Zn/3Al–Na (Figure 3B), where the band attributed to ν_3_ vibration is narrow and split into two bands at 1109 and 1156 cm^–1^ and with a shoulder at 1191 cm^–1^. This splitting is characteristic of the presence of sulfate in a distorted tetrahedral geometry. This is comprehensible since this sample has the highest crystallinity (Figure 2A-g). Similar behavior has already been reported for bimetallic phases of LDHs synthesized with different M^2+^/Al^3+^ compositions and intercalated with sulfate.^17,18^ In this sample, the bands at 548, 618, and 790 cm^–1^ are also more prominent.

The ICP-OES analyses of the Zn/Ni–Al–K (Table 3) and Zn/Ni–Al–K (Table 4) series indicated compositions close to those predicted in the syntheses (Tables 1 and 2). One sample with a similar basal distance and with the composition Zn_0.67_Cr_0.33_(OH)_2_]­[(SO_4_)_0.22_Na_0.11_·1.25H_2_O was already described years ago, but at that time, this composition had not been studied since LDHs were just considered to exchange anions.^29^

**Table 3:** Composition of Samples from the Ni/Zn–Al–K System, Obtained by ICP-OES^a^

system	Ni^2+^	Zn^2+^	Al^3+^	SO_4_^2–^	K^+^
6Ni/3Al–K	0.677	-	0.323	0.198	0.094
5Ni/1Zn–3Al–K	0.535	0.125	0.340	0.193	0.103
4Ni/2Zn–3Al–K	0.451	0.204	0.345	0.209	0.115
3Ni/3Zn–3Al–K	0.330	0.333	0.337	0.191	0.109
2Ni/4Zn–3Al–K	0.224	0.445	0.331	0.207	0.100
1Ni/5Zn–3Al–K	0.112	0.547	0.341	0.204	0.111
6Zn/3Al–K	-	0.662	0.338	0.210	0.103

a The ideal indices of the formulas are 1 = 0.111; 2 = 0.222; 3 = 0.333; 4 = 0.444; 5 = 0.555; 6 = 0.666; SO_4_^2–^ = 0.222; and K^+^ = 0.111.

**Table 4:** Composition of Samples from the Zn/Al–Na System, Obtained by ICP-OES^a^

system	Ni^2+^	Zn^2+^	Al^3+^	SO_4_^2–^	K^+^
6Ni/3Al–Na	0.665	-	0.335	0.235	0.112
5Ni/1Zn–3Al–Na	0.568	0.084	0.348	0.212	0.097
4Ni/2Zn–3Al–Na	0.491	0.157	0.352	0.216	0.091
3Ni/3Zn–3Al–Na	0.365	0.280	0.355	0.209	0.106
2Ni/4Zn–3Al–Na	0.244	0.407	0.349	0.219	0.090
1Ni/5Zn–3Al–Na	0.123	0.528	0.349	0.198	0.092
6Zn/3Al–Na	-	0.659	0.341	0.212	0.097

a The ideal indices of the formulas are 1 = 0.111; 2 = 0.222; 3 = 0.333; 4 = 0.444; 5 = 0.555; 6 = 0.666; SO_4_^2–^ = 0.222; and Na^+^ = 0.111.

In some cases, the content of sulfate is slightly smaller than the predicted value due to the possible presence of carbonate. Using the 3Ni/3Zn/3Al–K sample as an example, the composition can be written as [Ni_0.333_Zn_0.333_Al_0.333_(OH)_2_]­[K_0.111_(H_2_O)_0.666_(SO_4_)_0.191_(CO_3_)_0.031_]·0.666H_2_O or in integral form [Ni_3_Zn_3_Al_3_(OH)_18_]­[K­(H_2_O)_6_(SO_4_)_1.718_(CO_3_)_0.281_]·6H_2_O. The presence of carbonate in the FTIR spectra and EDS is not evidenced, probably due to the very small concentration. Another possibility to increase the amount of sulfate would be for the nickel to be partially oxidized to Ni^3+^, but even in this possible case, potassium would be expected to be absent. Based on these observations and the constancy of the potassium concentration in the samples, it can then be concluded that all the synthesized samples belong to the family of shigaite-like LDH.^9^

The EDS spectra (Figure 4A,B) confirmed the presence of the elements used and the tendency of the nickel signal to decrease and the zinc signal to increase throughout the series. Although semiquantitative, is it evidenced in the spectral sequence from a to g (Figure 4A,B) that nickel is progressively decreased while zinc is progressively increased. Despite the difficulty of identifying alkali metal cations due to the overlapping of the curves, no other elements could be detected, again indicating the purity of the samples.

**Figure 4:**
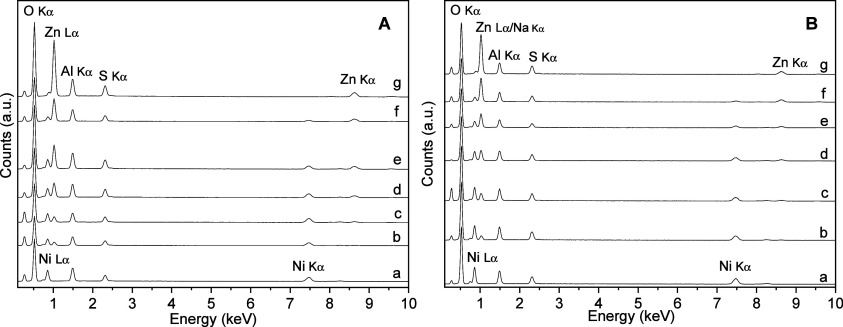
EDS spectra of the Zn/Ni–Al–K (A) and Zn/Ni–Al–K (B) system. 6Ni/3Al–K (A–a), 5Ni/1Zn–3Al–K (A-b), 4Ni/2Zn–3Al–K (A-c), 3Ni/3Zn–3Al–K (A-d), 2Ni/4Zn–3Al–K (A-e), 1Ni/5Zn–3Al–K (A-f), and 6Zn/3Al–3Al–K (A-g). 6Ni/3Al–3Al–Na (B-a), 5Ni/1Zn–3Al–Na (B-b), 4Ni/2Zn–3Al–Na (B-c), 3Ni/3Zn–3Al–Na (B-d), 2Ni/4Zn–3Al–Na (B-e), 1Ni/5Zn–3Al–Na (B-f) and 6Zn/3Al–Na (B-g).

Solely considering the simple intercalation of sulfate as occurs with the hydrotalcite-like LDH, the expected materials obtained in these syntheses would have the composition [Ni_6–*x*_Zn_*x*_Al_3_(OH)_18_]­[(SO_4_)_1.5_]·*n*H_2_O or Ni_1–*x*‑*y*_Zn_*y*_Al_*x*_(OH)_2_]­[(SO_4_)_*x*/2_]·*n*H_2_O. However, if the presence of carbonate is excluded, the amount of sulfate would equal the amount of aluminum divided by 2 and potassium would not be expected to be present. These are not the cases.

To be sure that potassium or sodium is not present as a contaminant, all of the samples were washed several times followed by centrifugation, and the slurry was redispersed in water. Consequently, the amounts of potassium and sodium as contaminants could be excluded.

The SEM images of the Zn/Ni–Al–K series, in general (Figure 5), showed submicrometric and highly aggregated particles.

**Figure 5:**
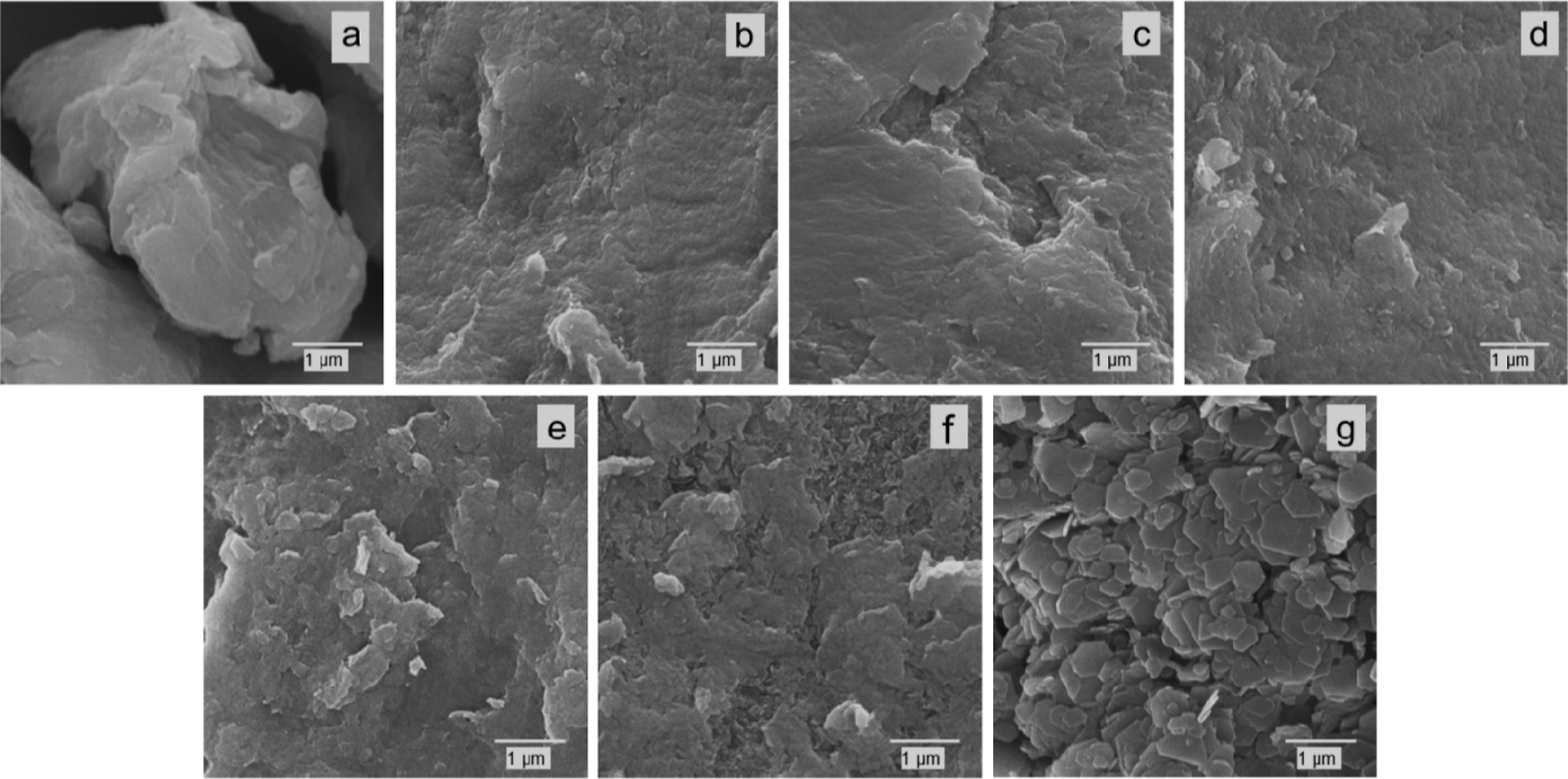
SEM images of the samples from the Zn/Ni–Al–K series: 6Ni/3Al (a), 5Ni/1Zn–3Al (b), 4Ni/2Zn–3Al (c), 3Ni/3Zn–3Al (d), 2Ni/4Zn–3Al (e), 1Ni/5Zn–3Al (f), and 6Zn/3Al (g).

The particles’ sizes tend to increase with the increase of zinc in the samples, culminating with the sample 6Zn/3Al (Figure 5g), where micrometric particles with platelet-like morphology typical of LDHs can be observed. This tendency is in accordance with the XRD patterns (Figure 1), which indicated a progressive increase in crystallinity and particle sizes in the series. Even when the particles are bigger (Figure 5g), the sizes are in the range of one micrometer with thicknesses in the nanometric range. Also, only in this sample is it possible to observe sharp corners close to 120°, suggesting hexagonal/trigonal symmetry of the unit cell.

The same tendency is also observed for the samples from the Zn/Ni–Al–Na system (Figure 6), in accordance with the XRD data (Figure 2). In general, the sizes of the particles along the basal planes are bigger in the Zn/Ni–Al–Na zinc-rich samples (Figure 6f,g) than in the same samples in the Zn/Ni–Al–K series (Figure 5f,g).

**Figure 6:**
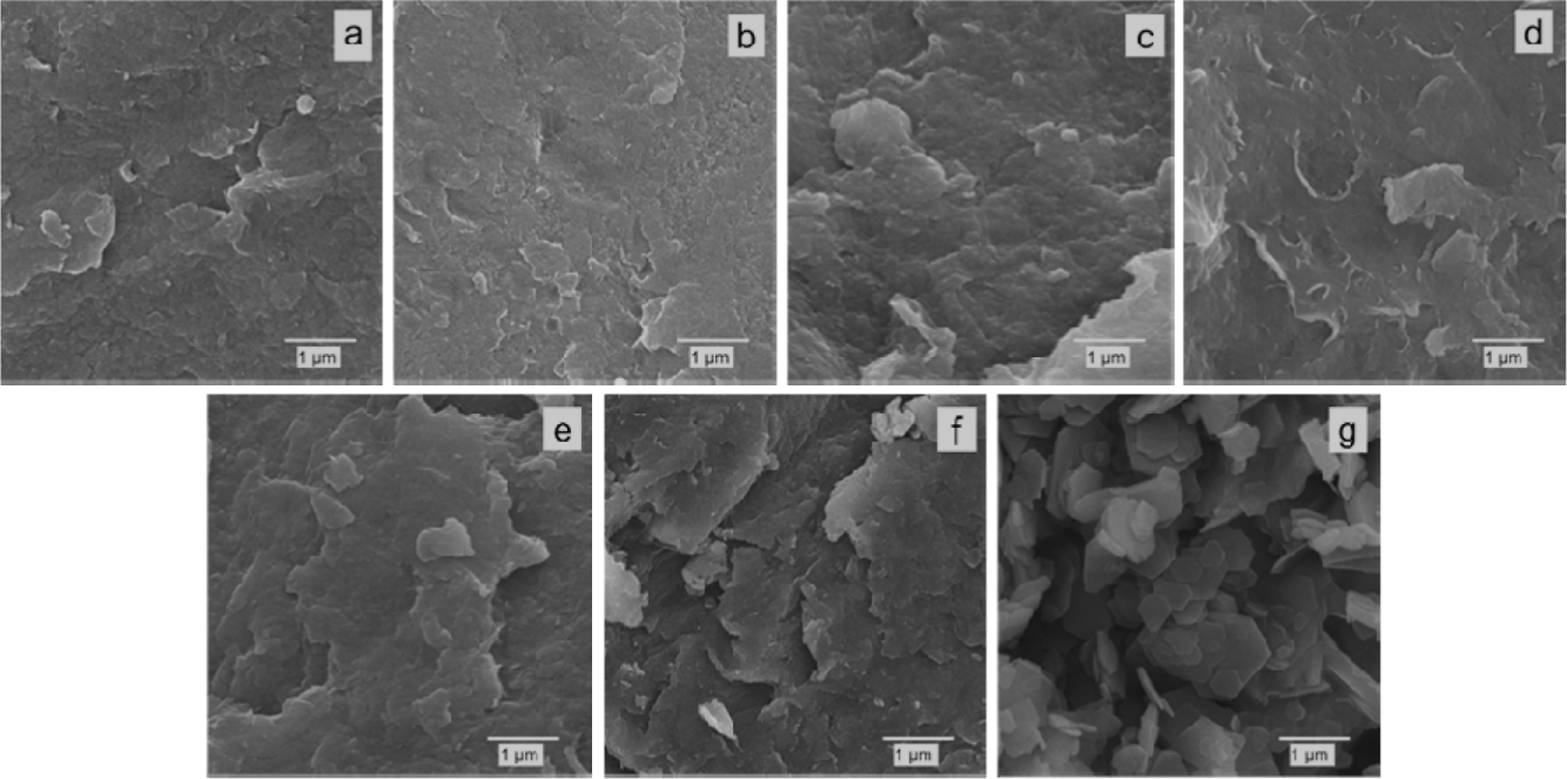
SEM images of the samples from the Zn/Ni–Al–Na series: 6Ni/3Al (a), 5Ni/1Zn–3Al (b), 4Ni/2Zn–3Al (c), 3Ni/3Zn–3Al (d), 2Ni/4Zn–3Al (e), 1Ni/5Zn–3Al (f), and 6Zn/3Al (g).

The thermogravimetric analysis (TGA) and derivative curves (DTG) of the Zn/Ni–Al–K series (Figure 7) indicated several events of mass loss from room temperature to 1000 °C.

**Figure 7:**
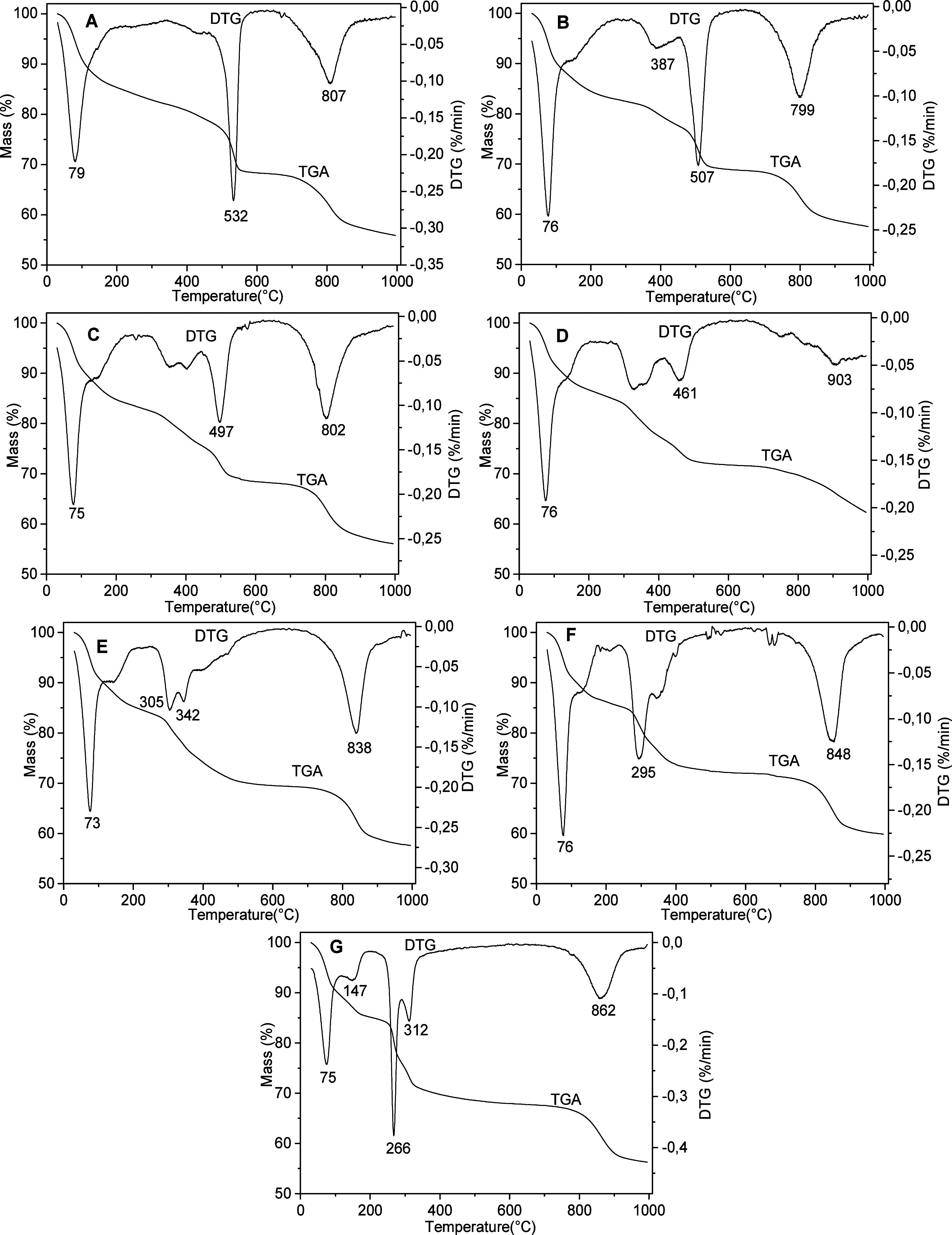
TGA/DTG curves of the samples from the Zn/Ni–Al–K series: 6Ni/3Al (A), 5Ni/1Zn–3Al (B), 4Ni/2Zn–3Al (C), 3Ni/3Zn–3Al (D), 2Ni/4Zn–3Al (E), 1Ni/5Zn–3Al (F), and 6Zn/3Al (G).

In general, the first mass loss in the range of room temperature to 200 °C occurs in one broad DTG peak centered in the range of 70–90 °C (sometimes in two separate peaks), attributed to removal of physiosorbed water molecules along with dehydration of intercalated sulfate and alkali metal cations. The second mass loss, in the range of 250–600 °C, is attributed to the dehydroxylation of the layers. Finally, the last event, from 600 to 1000 °C, is due to the partial metal sulfates’ decomposition and formation of the respective oxides/spinels.

With small shifts of the temperatures, the phase 6Zn/3Al–K decomposition profile (Figure 7G) is like the previously reported one for the sample synthesized under similar conditions, presenting DTG peaks at 75, 166, 290, and 815 °C.^31^

In general, the step attributed to the dehydroxylation of the layers is shifted to lower temperatures in the series 6Ni/3Al–K to 6Zn/3Al–K, while the DTG peaks can be observed at 532 °C for 6Ni/3Al (Figure 7A), 507 °C for 5Ni/1Zn–3Al (Figure 7B), 497 °C for 4Ni/2Zn–3Al (Figure 7C), 461 °C for 3Ni/3Zn–3Al (Figure 7D), 342 and 305 °C for 2Ni/4Zn–3Al (Figure 7E), 295 °C for 1Ni/5Zn–3Al (Figure 7F), and 266 and 312 °C for 6Zn/3Al (Figure 7G). As observed in the DTG peaks, decomposition of the metal sulfates occurs at similar temperatures for all samples.

As reported previously,^18,31^ even at the end of the analyses, the final mass is not constant, indicating that metal sulfates were not totally decomposed, explaining the slightly bigger experimental residual masses after the thermal treatment at 1000 °C in comparison with the theoretical values (Table 5). These differences can also be attributed to the difficulty of finding the exact point where the anhydrous phases are obtained and to the ICP-OES chemical analyses, which also produce some uncertainties.

**Table 5:** Theoretical and Experimental Masses of Residues of the Zn/Ni–Al–K Series after Thermal Treatment at 1000 °C

phase	formula^a^	theoretical residual mass (%)^b^	experimental residual mass (%)^b^	deviation^c^ (%)
6Ni/3Al	[Ni_0.677_Al_0.323_(OH)_2_][K_0.094_(SO_4_)_0.178_]	68.67	66.75	2.87
5Ni/1Zn	[Ni_0.535_Zn_0.125_Al_0.340_(OH)_2_][K_0.103_(SO_4_)_0.193_]	68.66	68.12	0.79
4Ni/2Zn	[Ni_0.451_Zn_0.204_Al_0.345_(OH)_2_][K_0.115_(SO_4_)_0.209_]	68.05	66.71	2.01
3Ni/3Zn	[Ni_0.330_Zn_0.333_Al_0.337_(OH)_2_][K_0.109_(SO_4_)_0.191_]	69.31	71.43	2.97
2Ni/4Zn	[Ni_0.224_Zn_0.445_Al_0.331_(OH)_2_][K_0.099_(SO_4_)_0.207_]	68.37	67.20	1.74
1Ni/5Zn	[Ni_0.112_Zn_0.547_Al_0.341_(OH)_2_][K_0.111_(SO_4_)_0.204_]	68.95	68.79	0.24
6Zn/3Al	[Zn_0.662_Al_0.338_(OH)_2_][K_0.103_(SO_4_)_0.210_]	67.26	66.22	1.57

a Based on the ICP-OES analysis.

b Based on the anhydrous phases producing the respective metal oxides/spinels and traces of metal sulfates.

c Deviation between the theoretical and the experimental data, based on anhydrous phases.

The anhydrous phases (based on the ICP-OES analysis) were obtained after the first DTG peak, and the small deviations between the experimental and theoretical residues (bigger in the experimental) are explained by the inconstancy of the mass present in the samples after 1000 °C, suggesting the presence of partially undecomposed metal sulfates.

The TGA/DTG curves of the samples from the Zn/Ni–Al–Na series are presented in the Supporting Information (Figure S1), and as examples of the Zn/Ni–Al–K, all the theoretical residual masses are smaller than the experimental ones after thermal treatment at 1000 °C, suggesting again traces of undecomposed metal sulfates (Table 6).

**Table 6:** Theoretical and Experimental Masses of Residues of the Zn/Ni–Al–Na Series after Thermal Treatment at 1000 °C

Phase	formula^a^	theoretical residual mass (%)^b^	experimental residual mass (%)^b^	deviation^c^ (%)
6Ni/3Al	[Ni_0.665_Al_0.335_(OH)_2_] [Na_0.112_(SO_4_)_0.235_]	63.79	64.17	0.60
5Ni/1Zn	[Ni_0.568_Zn_0.084_Al_0.348_(OH)_2_][Na_0.097_(SO_4_)_0.212_]	66.78	69.84	4.38
4Ni/2Zn	[Ni_0.491_Zn_0.157_Al_0.352_(OH)_2_][Na_0.091_SO_4_)_0.216_]	66.60	66.88	0.42
3Ni/3Zn	[Ni_0.365_Zn_0.280_Al_0.355_(OH)_2_][Na_0.106_(SO_4_)_0.209_]	67.49	68.93	2.08
2Ni/4Zn	[Ni_0.244_Zn_0.407_Al_0.349_(OH)_2_][Na_0.089_(SO_4_)_0.219_]	66.92	69.00	3.02
1Ni/5Zn	[Ni_0.123_Zn_0.528_Al_0.349_(OH)_2_][Na_0.092_(SO_4_)_0.198_]	68.48	69.84	1.95
6Zn/3Al	[Zn_0.659_Al_0.341_(OH)_2_][Na_0.097_(SO_4_)_0.212_]	71.86	74.32	3.31

a Based on the ICP-OES analysis.

b Based on the anhydrous phases producing the respective metal oxides/spinels and traces of metal sulfates.

c Deviation between the theoretical and the experimental data, based on anhydrous phases.

Again, the phase 6Zn/3Al–Na decomposition profile (Figure SI1–G) is similar to the previously reported data of the sample synthesized under similar conditions, presenting DTG peaks at 78, 161, 264, and 830 °C.^31^ The dihydroxylation of the layers’ temperatures is also shifted to lower values than the series from 6Ni/3Al to 6Zn/3Al (Figure SI1).

Since more than two metals are present in the new LDHs, unexpected properties can be expected in comparison with the traditional LDH of the hydrotalcite family. After calcination at different temperatures, the crystallinity degree, particle shapes and sizes, surface area, and porosity can be controlled. Also, the binary or ternary mixed oxides, especially in the form of spinels, can present new properties, broadening the applications in different fields of science and technology.^32^

In the case of Na-shigaite, Na-natroglaucocerinite, and Na-motukoreaite, due to the variation of colors (in the case of Na-shigaite, from red to orange, yellow, brown, and black) and genesis, it is possible that in the future, other minerals of the same family will be discovered containing not only sodium but other alkali metals or alkaline earth metal cations, intercalated between the layers. Even two alkali metal cations are expected to be intercalated/cointercalated as observed experimentally.

It is also important to mention that until now, no bimetallic or trimetallic layered double hydroxide mineral intercalated with sulfate and potassium has been discovered. It is highly likely that these minerals equivalent to the synthetic ones will be discovered in the future, not only involving the system Ni/Zn–Al but also other synthetic phases described recently.^16–21^

In an attempt to perform the ion exchange reaction between [Zn_6_Al_3_(OH)_18_]­[Na­(H_2_O)_6_(SO_4_)_2_]·6H_2_O with nitrate ions, the resulting compound presented the formula [Zn_6_Al_3_(OH)_18_]­[(NO_3_)_3_·*n*H_2_O, demonstrating that the [Na­(H_2_O)_6_(SO_4_)_2_]^3–^ clusters were removed, having been replaced by nitrate ions, resulting in a hydrotalcite-like LDH (not shown), just as observed in previous experiments.^16^ The same tendency is expected for the other compounds in the series.

## Conclusions

Bimetallic and trimetallic layered double hydroxides with the composition [Ni_6–*x*_Zn_*x*_Al_3_(OH)_18_]­[A­(H_2_O)_6_(SO_4_)_2_]·6H_2_O (*x* from 0 to 6; A = Na^+^ or K^+^) were synthesized by coprecipitation with increased pH and characterized by several instrumental techniques.

X-ray diffraction (XRD) indicated the prevalence of basal diffraction peaks with basal distances close to 11 Å, typical of LDHs intercalated with sulfate in double -layer arrangement together with alkali metal cations, both hydrated.

Fourier-transform infrared spectroscopy (FTIR) indicated the presence of typical series of bands, attributed to S–O (from sulfate), M–O (M = metal of the layers), and O–H (from water molecules and hydroxide layers). Sulfate bands are split due to the distortion from the tetrahedral geometry as well as the interaction with water molecules, cations, and anionic charged layers.

Scanning electron microscopy (SEM) indicated the typical platelet-like morphology of LDHs, with micrometer-aggregated particles having nanometric thicknesses. In both Zn/Ni–Al series, the particle sizes tended to increase with the replacement of nickel with zinc in the samples, culminating with the bigger particles in the samples 6Zn/3Al-A (A = Na^+^ or K^+^). This tendency was in accordance with the XRD patterns, which indicated a progressive increase of crystallinity and particle sizes in the series.

Energy-dispersive X-ray spectroscopy (EDS) spectra indicated a homogeneous distribution of the metal cations in the particles and the stoichiometry close to that used in the synthesis, without the presence of impurities.

Inductively coupled plasma-optical emission spectroscopy (ICP-OES) confirmed the compositions of the materials, which were remarkably close to ideal values, suggesting the absence of simultaneous precipitation of other materials (especially hydroxides).

The thermogravimetric analyses (TGA) and differential (DTG) curves indicated several mass loss events from room temperature to 1000 °C, attributed to the removal of physiosorbed water molecules, dehydration of sulfate and alkali metal cations, dehydroxylation of the layered structure, and partial metal sulfates’ decomposition, with the formation of the respective oxides/spinels.

We believe that investigating new compounds in these series will contribute to future applications, as in examples recently published for synthesized LDHs by our research group, such as the stabilization of Pickering emulsions,^33,34^ emulsions with antimicrobial activity,^35^ sensors to detect uric acid and dopamine, ascorbic acid, glucose and carbedazim,^36,37^ photocatalytic hydroxylation of terephthalic acid, and photodegradation of sodium diclofenac^38^ as well as dye adsorption.^39^ Several other investigations of potential applications are under way and will be the subject of forthcoming publications.

## Supplementary Material



## Notes

TGA/DTG curves of the samples from the Zn/Ni–Al–Na series (PDF)
