# Supplementary material for: Novel Bimetallic and Trimetallic Layered Double Hydroxides with the Compositions [Ni6‑xZn x Al3(OH)18][A(H2O)6(SO4)2]·6H2O (x from 0 to 6; A= Na+ o K+)
Source: ACS Omega. 2026 Mar 20;11(12):19052–62. doi: 10.1021/acsomega.5c11618 (PMC13044832; doi:10.1021/acsomega.5c11618)
Supplement: Supplementary file 1 [file ao5c11618_si_001.pdf]

Novel bimetallic and trimetallic layered double hydroxides with the compositions $[Ni_{6-x}Zn_xAl_3(OH)_{18}][A(H_2O)_6(SO_4)_2]\cdot 6H_2O$ (x from 0 to 6; A= Na^+ o K^+).

Anne Raquel Sotiles[a], Marco Tadeu Grassi[a], Mayara Padovan dos Santos[a], Gabriel Kavilhuka Metzger[b], Fernando Wypych[a,b,c*]

Federal University of Paraná - [a]Department of Chemistry, [b]Post-Graduation Program in Engineering and Materials Science (PIPE). CP 19032, Centro Politécnico, Jardim das Américas - 81531-980 - Curitiba - PR - Brazil.
Federal University of Technology - [c]Post-Graduation Program in Materials Science and Engineering (PPGCEM) – CEP 86036-370 - Londrina - PR - Brazil.

Figure S1 - TGA/DTG curves of samples from the Zn/Ni-Al-Na series.

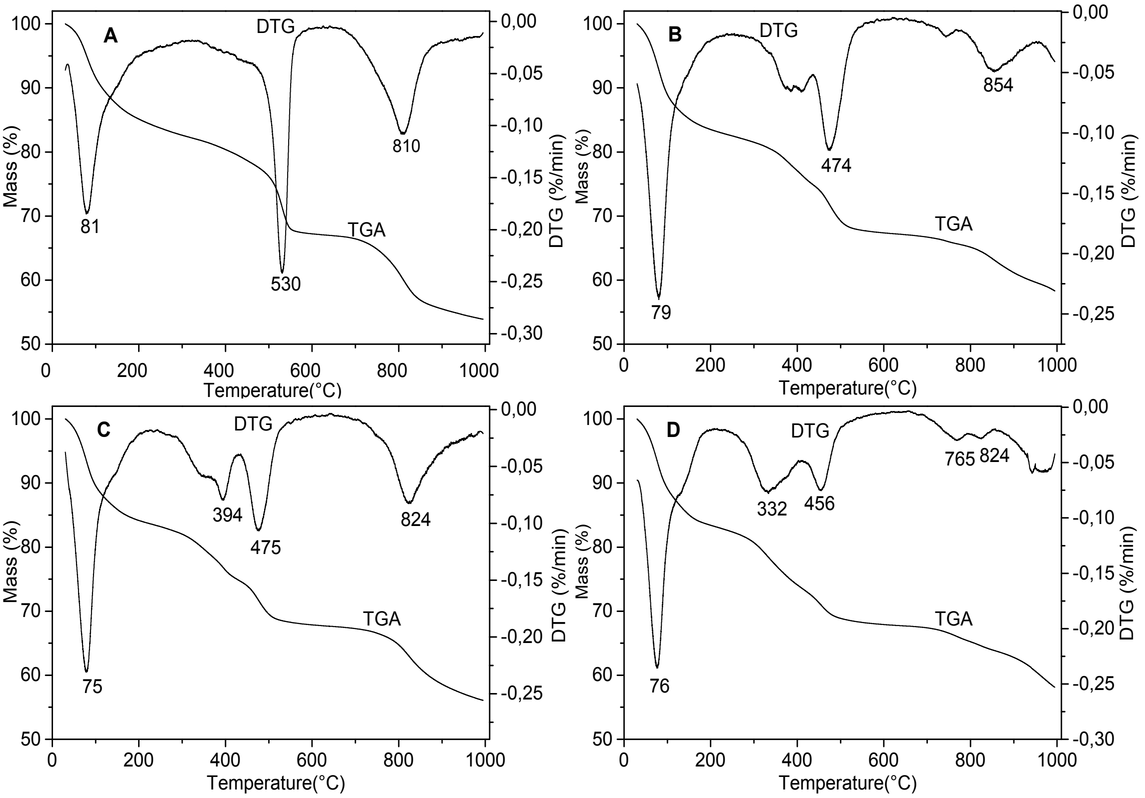

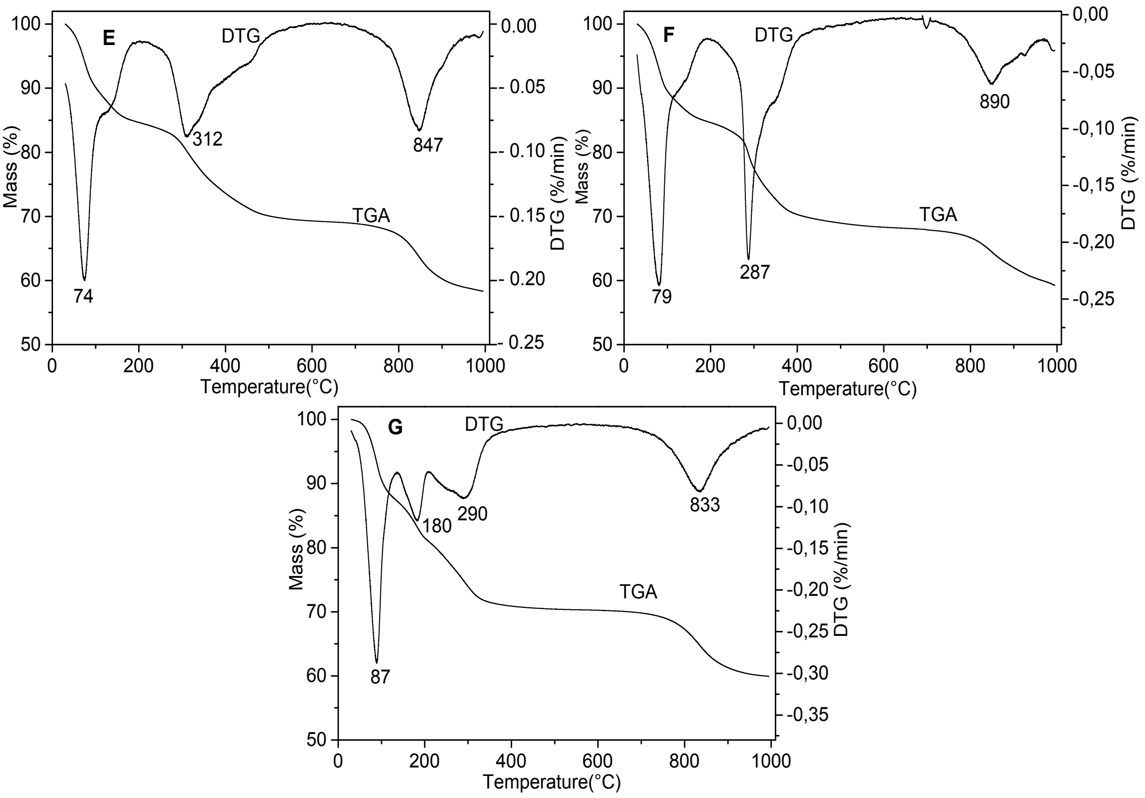
E
DTG
TGA
74
312
847
Mass (%)
DTG (%/min)
Temperature(°C)
F
DTG
TGA
79
287
890
Mass (%)
DTG (%/min)
Temperature(°C)
G
DTG
TGA
87
180
290
833
Mass (%)
DTG (%/min)
Temperature(°C)
